# An *N*-Halamine/Graphene Oxide-Functionalized Electrospun Polymer Membrane That Inactivates Bacteria on Contact and by Releasing Active Chlorine

**DOI:** 10.3390/polym13162784

**Published:** 2021-08-19

**Authors:** Shi Lan, Jinghua Zhang, Jie Li, Yanan Guo, Xianliang Sheng, Alideertu Dong

**Affiliations:** 1 College of Science, Inner Mongolia Agricultural University, Hohhot 010018, China; imaushilan@126.com (S.L.); Z2101800672@163.com (J.Z.); 15848106457@163.com (J.L.); gynxddyx@163.com (Y.G.); 2 College of Chemistry and Chemical Engineering, Inner Mongolia University, Hohhot 010021, China

**Keywords:** *N*-halamine, graphene oxide, antibacterial material, fibrous membrane, on contact, by releasing

## Abstract

The emergence of antibiotic-resistant “superbugs” in recent decades has led to widespread illness and death and is a major ongoing public health issue. Since traditional antimicrobials and antibiotics are in many cases showing limited or no effectiveness in fighting some emerging pathogens, there is an urgent need to develop and explore novel antibacterial agents that are both powerful and reliable. Combining two or more antibiotics or antimicrobials has become a hot topic in antibacterial research. In this contribution, we report on using a simple electrospinning technique to create an *N*-halamine/graphene oxide-modified polymer membrane with excellent antibacterial activity. With the assistance of advanced techniques, the as-obtained membrane was characterized in terms of its chemical composition, morphology, size, and the presence of active chlorine. Its antibacterial properties were tested with *Escherichia coli* (*E. coli*) as the model bacteria, using the colony-counting method. Interestingly, the final *N*-halamine/graphene oxide-based antibacterial fibrous membrane inactivated *E. coli* both on contact and by releasing active chlorine. We believe that the synergistic antimicrobial action of our as-fabricated fibrous membrane should have great potential for utilization in water disinfection, air purification, medical and healthcare products, textile products, and other antibacterial-associated fields.

## 1. Introduction

Infectious diseases arising from pathogenic bacteria have become one of the leading causes of death worldwide, especially with the emergence of antibiotic resistance in pathogenic bacteria [1,2,3]. Antibiotic resistance—multidrug-resistance being the most serious—is threatening our ability to treat infectious diseases and has become a burden on global economies [4,5]. It has been widely recognized that the emergence of antibiotic resistance in pathogenic bacteria is largely due to the misuse and overuse of antibiotics, resulting in the increasing failure of traditional antibiotics [6,7]. This situation is propelling an urgent search for new antibiotics that act in different ways from traditional ones and thereby can overcome bacterial resistance [8].

Many antibacterial agents that effectively kill pathogenic bacteria have been discovered to be successful in combating pathogenic bacteria-associated infectious diseases [9,10,11]. To date, numerous chemicals and biomolecules, such as guanidine, *N*-halamine, povidone-iodine, peptide, and others, have been developed as biocides, and some have been commercialized. Of the effective antibacterial agents listed above, *N*-halamines have attracted significant research interest due to their powerful antibacterial activity against a wide spectrum of pathogenic bacteria, as well as their long-term stability in harsh conditions, high durability, regenerability upon exposure to household bleaching cycles, low toxicity, and low cost [12,13]. At present, the use of *N*-halamines ranges from water disinfection to air purification, food packaging and storage, medical and healthcare products, and textiles [14]. Notably, unlike some antibacterial agents that kill bacteria via a single pathway, *N*-halamines can transfer an active halogen to bacterial receptors on contact or by releasing the active halogen (e.g., Cl^+^) [15]. Such a contact/release combined antibacterial mode enables *N*-halamines to be active in both wet and dry environments [16].

In recent years, nanomaterials have offered promising alternatives for antibacterial therapy because many have reported that they are less prone to form antibiotic resistance in pathogenic bacteria [17]. Previous reports have demonstrated that several nanomaterials, such as silver nanoparticles (Ag NPs), gold nanorods (Au NRs), zinc oxide nanoflowers (ZnO NFs), polymer fibers (PNFs), carbon nanotubes (CNTs), graphene oxide (GO), graphdiyne oxide (GDYO), black phosphorus (BP), and others, are successful in eradicating bacteria without causing bacterial resistance [5,18,19,20]. Of these examples, GO has emerged as a promising nanoagent for bacterial elimination because of its unique features, such as a larger specific surface area and higher activity compared to its bulk counterparts [21]. So far, numerous scientists and engineers have devoted their efforts to discovering the antibacterial mechanisms of GO nanosheets [22]. Basically, the mechanism involves two main modes: (i) physical damage induced by direct contact between the sharp edges of GO nanosheets and bacteria, and (ii) oxidative stress induced by reactive oxygen species (ROS). Some scientists promote the former mode, while others support the latter. A few endorse other mechanisms, including wrapping or trapping bacterial membranes, extracting lipid bilayers, interfering with protein–protein interactions, and self-killing mechanisms [22].

In clinical use, combining two or more active antibiotics (or antimicrobials) is an effective therapy for counteracting bacteria without causing antibiotic resistance [23]. It has become clear that synergistic therapy is superior to individual approaches. Vasan’s group found that a ZnO/Ag hybrid showed higher antibacterial activity against Gram-positive and Gram-negative bacteria than its individual components (i.e., ZnO and Ag) [24]. In Patir’s report, the excellent photothermal antibacterial and antibiofilm capabilities of BP/Au nanocomposites destroyed the bacterial cell membrane more efficiently than pristine BP [25]. Owing to its ease of operation and low cost, broad-spectrum effectiveness, the electrospinning technique has gained a great achievement on the combination of two or more active antibacterial agents for fighting pathogens [26]. Since the term electrospinning was defined in mid 1990s, numerous antibacterial nanomaterials have been synthesized [27,28,29,30]. To date, several published reviews of electrospinning have focused on some aspects of synthesis technology, characterization, antibacterial property, and biomedical applications [31,32,33,34,35].

In this contribution, we used a one-step facile electrospinning strategy to fabricate a fibrous membrane containing 1,3-dichloro-5,5-dimethylhydantoin (DCDMH, a typical *N*-halamine) and graphene oxide (GO), with polyacrylonitrile (PAN) as the support matrix. The as-obtained product PAN/GO/DCDMH was characterized by scanning electron microscopy (SEM), transmission electron microscopy (TEM), energy-dispersive X-ray spectroscopy (EDX), Fourier transform infrared spectroscopy (FTIR), X-ray photoelectron spectroscopy (XPS), and iodometric/thiosulfate assay. The synthesis of the PAN/GO/DCDMH fibrous membrane was regulated by tuning the feed ratio of PAN to GO and to DCDMH. After a series of antibacterial evaluations, the as-obtained fibrous membrane showed excellent antibacterial activity against *Escherichia coli* (*E. coli*) on contact and by releasing active chlorine. Such a contact-release synergy may be an efficient tactic for addressing severe antibiotic resistance caused by superbugs. We believe this work provides a guide for exploring advanced antibacterial materials that possess great potential for broad-spectrum antibacterial applications in water and air disinfection, medical and healthcare product, textile product, and other fields.

## 2. Materials and Methods

### 2.1. Materials

DCDMH was purchased from Aladdin Industrial Inc. Sodium nitrate, potassium permanganate, H_2_O_2_ (30 wt%), ethanol (EtOH), and *N,N*-dimethylformamide (DMF) were purchased from Sinopharm Chemical Reagent Co., Ltd. PAN was obtained from the Tianjin Chemical Reagent Plant. Natural graphite powder was obtained from Nanjing XFNANO Materials Tech Co., Ltd. Deionized water supplied by a Millipore system (Millipore Inc., MA, USA) was utilized for all of the experiments. All chemicals were used without purification.

### 2.2. Synthesis of Graphene Oxide

Graphene oxide (GO) was synthesized according to Hummers’ method [36]. About 1.0 g of commercial graphite powder was stirred in 30 mL of cold concentrated sulfuric acid for 1 h, and 1.2 g of sodium nitrate was then introduced to the suspension. Subsequently, 3.0 g of potassium permanganate was added gradually with stirring at 20 °C, then the mixture was stirred at 40 °C for 6 h. After that, 40 mL of distilled water was slowly added to the mixture and stirred at 98 °C for 15 min. The reaction was terminated by adding 150 mL of distilled water and 5 mL of H_2_O_2_ (30 wt%). After the suspension was dialyzed for one week, the GO was obtained. Then GO nanosheets were exfoliated from the crude GO by sonication for 12 h.

### 2.3. Synthesis of PAN/GO/DCDMH Fibrous Membrane

Antibacterial fibrous membrane was synthesized using an electrospinning technique [37]. Specifically, a mixture of 0.15 g of DCDMH, 0.05 g of GO, and 1.15 g of PAN was dissolved in DMF to make a quaternary mixture, then stirred overnight to obtain a beige, transparent, and viscous electrospinning precursor solution. This mixture was spun on an electrospinning unit with a high voltage of 12 kV at 25–28 °C to obtain the PAN/GO/DCDMH fibrous membrane. To confirm the antibacterial mechanism, PAN, PAN/GO, PAN/DCDMH, PAN/GO_0.1_/DCDMH, PAN/GO_0.15_/DCDMH, PAN/GO/DCDMH_0.10_, and PAN/GO/DCDMH_0.20_ fibrous membranes were prepared as comparative materials by tuning the feed ratios. Table 1 presents the feed ratios of PAN, GO, and DCDMH to DMF in the synthesis of PAN/GO/DCDMH.

### 2.4. Characterization

SEM (Shimadzu, Co. Ltd., Kyoto, Japan) was carried out on a Shimadzu SSX-550 field emission scanning electron microscope. A drop of the sample suspended in ethanol was placed on a piece of silicon wafer. After 10 min of air-drying, a thin gold coating was applied to prevent charging during scanning, then a thorough microscopic study was carried out. EDX spectroscopy was also used during the SEM measurements to examine the elemental composition of the samples. The detailed morphology, size, and inner state of the samples were surveyed using a Hitachi H-8100 transmission electron microscope (Hitachi, Ltd., Tokyo, Japan). For TEM imaging, the samples were suspended in ethanol with the assistance of sonication at 35 KHz, and the as-prepared suspensions were added onto a copper grid and air-dried at room temperature. FTIR spectrometry was performed on a Thermo Nicolet Avatar 370 FTIR spectrometer (Thermo Fisher Scientific Co. Ltd., Waltham, MA, USA). XPS was run on a PHI-5000CESCA system with Mg Kα radiation. The active chlorine content was quantified using an iodometric/thiosulfate assay.

### 2.5. Antibacterial Assay

The sample suspensions were challenged with *E. coli* 8099, a typical Gram-negative bacterium, using the colony-counting method [38]. First, *E. coli* were grown overnight at 37 °C in Luria−Bertani medium (LB, 10 g of tryptone and 5 g of yeast extract/liter), and the bacterial cells were harvested by centrifugation, washed with phosphate-buffered saline, and diluted to concentrations of 1 × 10^5^ CFU∙mL^−1^. Then 50 μL of bacteria suspension was mixed with 0.45 mL of sample suspension (100 mg∙mL^−1^) and incubated under constant shaking (200 rpm). After a certain period of contact time, the mixture was serially diluted, and 100 μL of each dilution was dispersed onto Luria−Bertani (LB) growth medium. Survival colonies on LB plates were counted after incubation for 24–36 h at 37 °C. The colony-counting tests were carried out in triplicate. The antibacterial rate was calculated according to the equation below:

Antibacterial rate (%)=(A−B/A)×100%

where *A* is the number of original cells and *B* is the number of survival cells.

### 2.6. Dialysis Test

The dialysis assay was performed according to Ahmed’s report [39]. The sample suspension was added into a dialysis bag (molecular weight cutoff 200 Da) and immersed in PBS at 37 °C in a constant temperature shaker for about two weeks. To examine the release action of active chlorine, the dialysate solution was examined using iodometric/thiosulfate titration. The antibacterial activity of the dialysate solution was studied using the colony-counting method. The release killing tests were performed in triplicate.

## 3. Results

**Figure 1:**
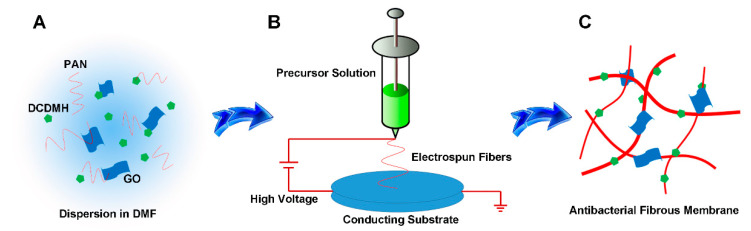
Schematic illustrating the synthesis of PAN/GO/DCDMH fibrous membrane using electrospinning: (**A**) dispersion of PAN, DCDMH, and GO in DMF; (**B**) electrospinning process; and (**C**) diagrammatic sketch of the PAN/GO/DCDMH nanostructures.

**Figure 2:**
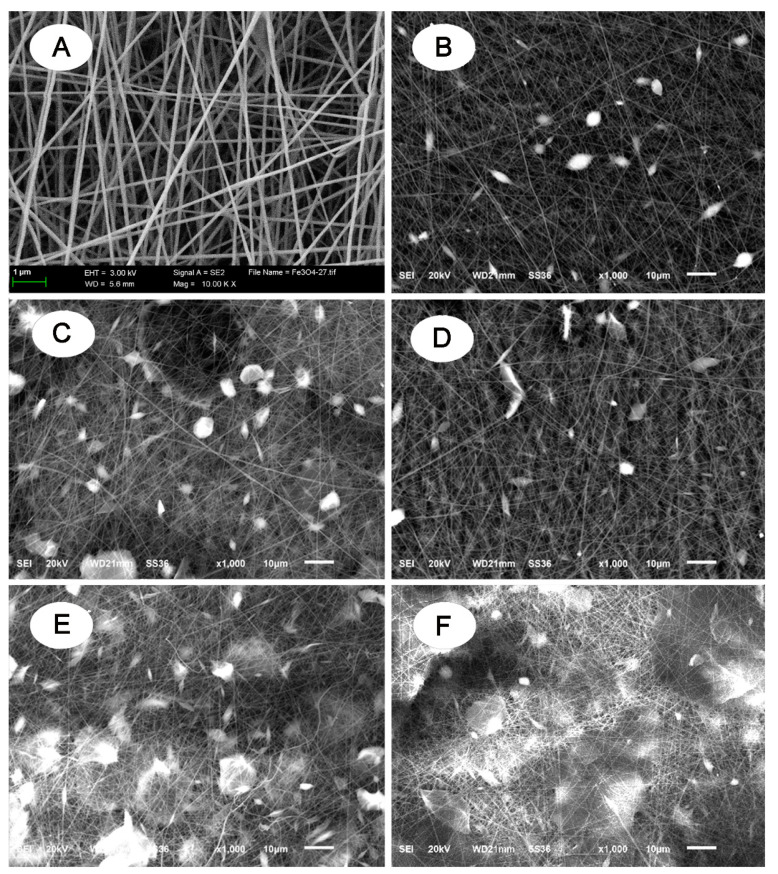
SEM images of (**A**) PAN, (**B**) PAN/DCDMH, (**C**) PAN/GO, (**D**) PAN/GO_0.05_/DCDMH, (**E**) PAN/GO_0.1_/DCDMH, and (**F**) PAN/GO_0.15_/DCDMH.

**Figure 3:**
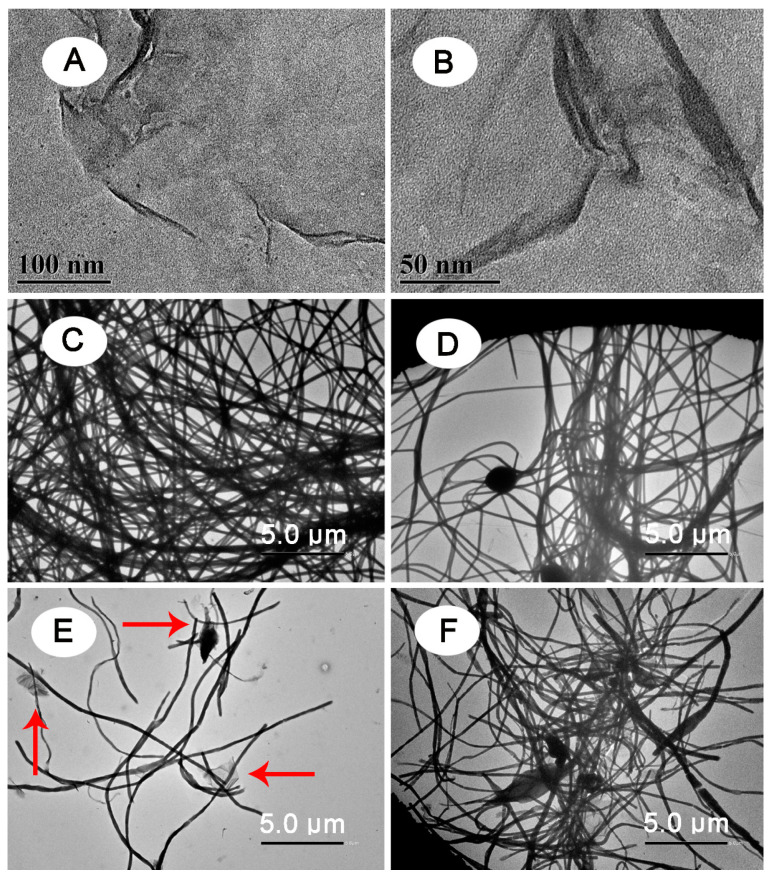
TEM image of (**A**,**B**) GO, (**C**) PAN, (**D**) PAN/DCDMH, (**E**) PAN/GO, and (**F**) PAN/GO/DCDMH.

Antibacterial fibrous membrane was synthesized using an electrospinning technique. Figure 1 illustrates the synthesis procedure for the PAN/GO/DCDMH fibrous membrane. The morphology, size, and surface state of the as-electrospun products were examined using SEM (Figure 2) and TEM (Figure 3). Figure 3A,B show that GO nanosheets with thin, sheet-like morphology were generated by Hummers’ method. As shown in Figure 2A, the electrospun PAN displayed randomly oriented, straight, and continuous features. Figure 3C demonstrates the uniform distribution of PAN; no aggregations are visible inside the fibers, demonstrating the homogeneity of the PAN matrix in the fiber system. In contrast, the DCDMH-containing (Figure 2B and Figure 3D) and GO-containing (Figure 2C and Figure 3E) PAN fiber presented much coarser surfaces. The GO-containing PAN fiber in particular showed many nanosheets (red arrows in Figure 3E) dispersed onto the PAN matrix because of the distribution of GO nanosheets among the PAN polymeric chains. These findings further confirmed the successful formation of PAN/DCDMH and PAN/GO nanostructures via electrospinning. When DCDMH and GO were mixed with PAN, followed by electrospinning, the as-obtained products (Figure 2D and Figure 3F) exhibited a fiber-based morphology coupled with well-dispersed nanosheets on the fibrous membrane. As for the fibrous membrane, its antibacterial activity is dependent on its morphology, size, surface state, and dispersibility of DCDMH and GO in the PAN supporting matrix.

We next examined the synthetic controllability of the PAN/GO/DCDMH nanostructures by tailoring the feed ratio of the reagents. The GO content in the electrospinning precursor solution was varied from 0.05 to 0.10, then to 0.15 g, while all other parameters remained fixed. Figure 2D–F shows that the products exhibited different surface appearances when the GO content in the electrospinning precursor solution was changed. The GO nanosheet loading rose as the GO content in the electrospinning precursor solution increased, indicating that the surface of the fibrous membrane became rough. Obviously, when the GO content was 0.05 g, comparatively little GO was loaded onto the fibrous membrane. The GO loadings became more obvious as the GO content increased to 0.10 and 0.15 g. Notably, when the GO content was 0.15 g, the GO nanosheets were completely scattered throughout the PAN polymeric matrix, appearing across the whole SEM image with a combined fiber–sheet morphology and a high GO loading. Hence, we were able to conclude that the morphology of the PAN/GO/DCDMH nanostructures could be regulated by tuning the GO content in the electrospinning precursor solution.

**Figure 4:**
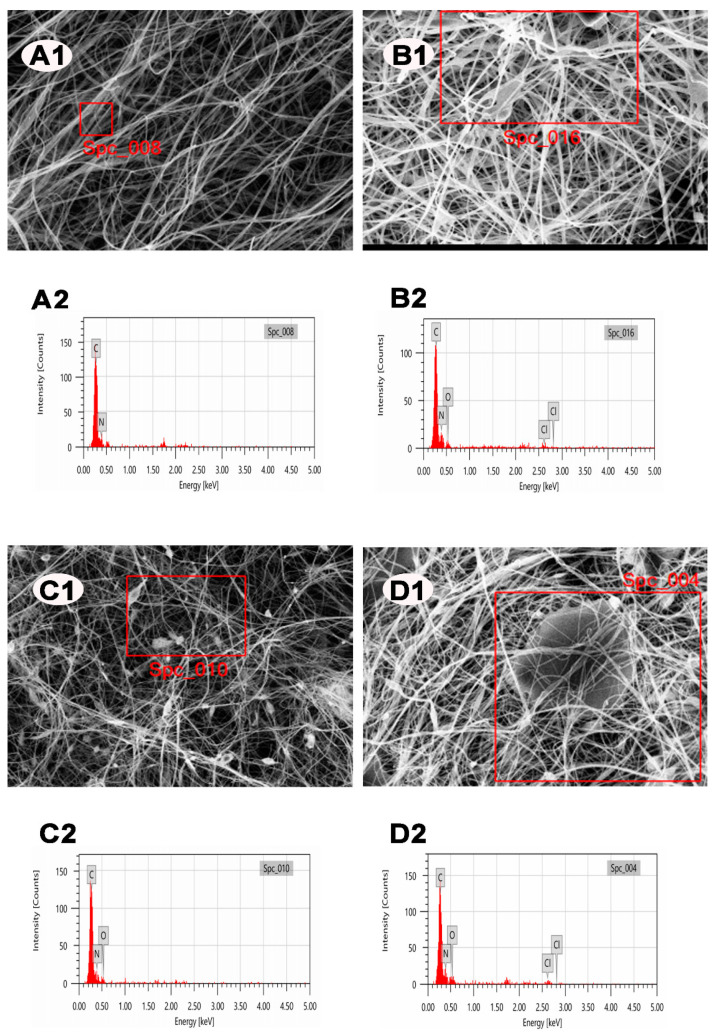
(**A1**,**B1**,**C1**,**D1**) SEM images and (**A2**,**B2**,**C2**,**D2**) EDX spectra of (**A**) PAN, (**B**) PAN/DCDMH, (**C**) PAN/GO, and (**D**) PAN/GO/DCDMH.

To acquire compositional information, the chemical compositions corresponding to the morphology of the as-electrospun products were tested using the SEM-EDX technique. Figure 4 and Table 2 present (A1–D1) SEM images and the corresponding (A2–D2) EDX spectra of (A) PAN, (B) PAN/DCDMH, (C) PAN/GO, and (D) the PAN/GO/DCDMH fibrous membrane. The EDX spectra were examined at selected regions on the SEM images. Pristine PAN (Figure 4(A2)) displayed two intensive peaks assigned to the C and N signals, suggesting that the PAN support consisted mainly of carbon and nitrogen. Unlike pristine PAN, PAN/DCDMH (Figure 4(B2)) presented two additional peaks, attributed to O and Cl, indicating the presence of hydantoin-based *N*-halamine on the surface of PAN and the successful combining of PAN and DCDMH. As can be seen in Figure 4(C2), the EDX spectrum of PAN/GO displayed elemental C, N, and O signals within the selected SEM region, demonstrating that PAN was well-combined with the GO nanosheets. The PAN/GO/DCDMH fibrous membrane (Figure 4(D2)) exhibited four typical signals corresponding to C, N, O, and Cl, suggesting the successful combination of PAN, DCDMH, and GO into the fiber system using electrospinning.

**Figure 5:**
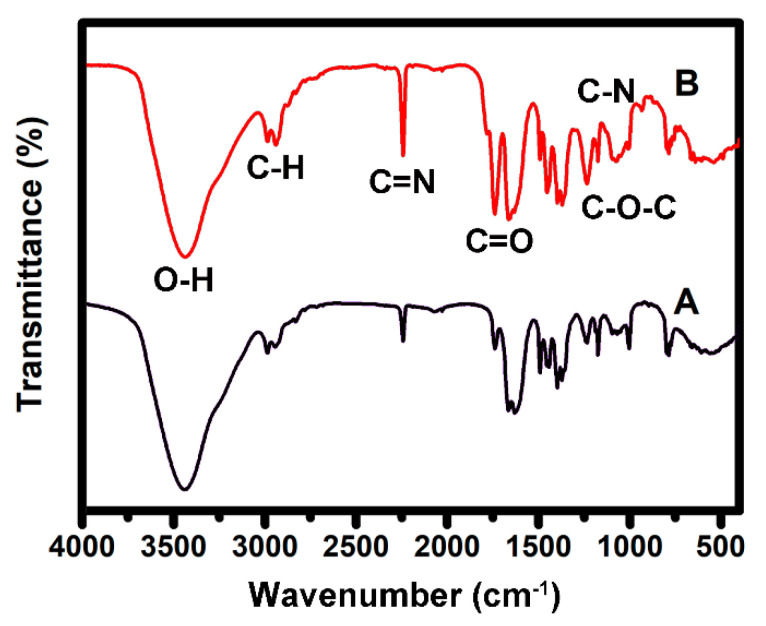
FTIR spectra of (**A**) PAN and (**B**) PAN/GO/DCDMH.

To further confirm the fabrication of the PAN/GO/DCDMH fibrous membrane, we performed FTIR spectroscopic analysis to record the characteristic groups. Figure 5 presents the FTIR spectra of pristine PAN and the PAN/GO/DCDMH fibrous membrane. The membrane clearly shows four characteristic vibrations of the C–H, C≡N, O–H, C–O–C, C=O, and C–N bands at around 2981, 2936, 2246, 3397, 1070–1170, 1734, and 1223 cm^–1^, respectively. The C–H and C≡N stretching vibrations are attributed to the PAN matrix [40,41], indicating that the as-electrospun product was supported by PAN polymeric chains. The appearance of the O–H, C–O–C, and C=O stretching vibrations confirmed the existence of GO [42,43]. In particular, the strong peak corresponding to the C–O–C and C=O vibrations further verified the high oxidation state on the GO nanosheets’ surface [43]. The stretching vibration of the C–N band suggested the presence of DCDMH in the nanostructures [44,45]. All these FTIR characteristic peaks fully demonstrated the successful combination of PAN, DCDMH, and GO into the fiber system through the one-step electrospinning strategy.

**Figure 6:**
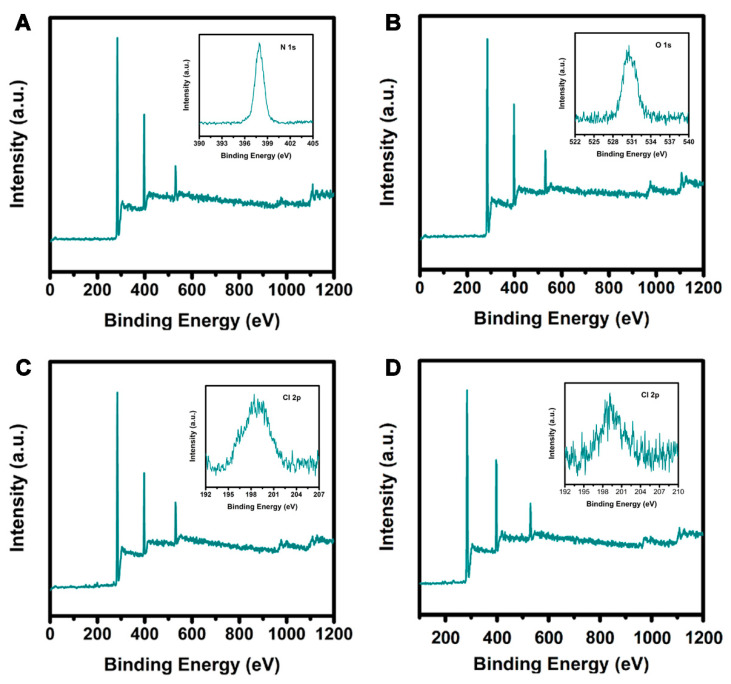
XPS survey scans, (insert in **A**) N 1s spectrum, (insert in **B**) O 1s spectrum, and (insert in **C**,**D**) Cl 2p spectrum of (**A**) PAN, (**B**) PAN/GO, (**C**) PAN/DCDMH, and (**D**) PAN/GO/DCDMH.

Detailed data about the chemical composition of the PAN/GO/DCDMH fibrous membrane were obtained from XPS spectra. Figure 6 presents XPS survey scans, showing the C 1s spectrum, O 1s spectrum, N 1s spectrum, and Cl 2p spectrum of (A) PAN, (B) PAN/GO, (C) PAN/DCDMH, and (D) PAN/GO/DCDMH. The pristine PAN (Figure 6A) exhibited two main peaks, C 1s and N 1s, indicating the PAN was made up of elemental carbon and nitrogen [46]. The appearance of C 1s at 284 eV and N 1s at 398 eV thus marks the existence of PAN as the support matrix for DCDMH and GO loading. The appearance of O 1s at 531 eV is attributed the glass substrate for sample immobilization. After the PAN and GO nanosheets were combined, the as-obtained PAN/GO (Figure 6B) exhibited three main peaks corresponding to C 1s, N 1s, and O 1s. The appearance of O 1s evidenced that GO had been added into the PAN matrix [47]. The O 1s is assigned to the carboxyl (-COOH), hydroxy (-OH), and epoxy (C–O–C) groups on the surface of the GO nanosheets. In the case of PAN/DCDMH (Figure 6C), the four typical peaks of C 1s, O 1s, N 1s, and Cl 2p were observed [48], indicating the successful synthesis of PAN/DCDMH. In particular, the formation of C 1s and Cl 2p peaks demonstrated the existence of the *N*-halamine structure in the electrospun fibrous membrane. When DCDMH and GO were mixed with PAN in DMF, then subjected to electrospinning (Figure 6D), the as-electrospun PAN/GO/DCDMH fibrous membrane presented the four characteristic peaks of C 1s, O 1s, N 1s, and Cl 2p, confirming the successful formation of the fibrous membrane using the electrospinning technique.

**Figure 7:**
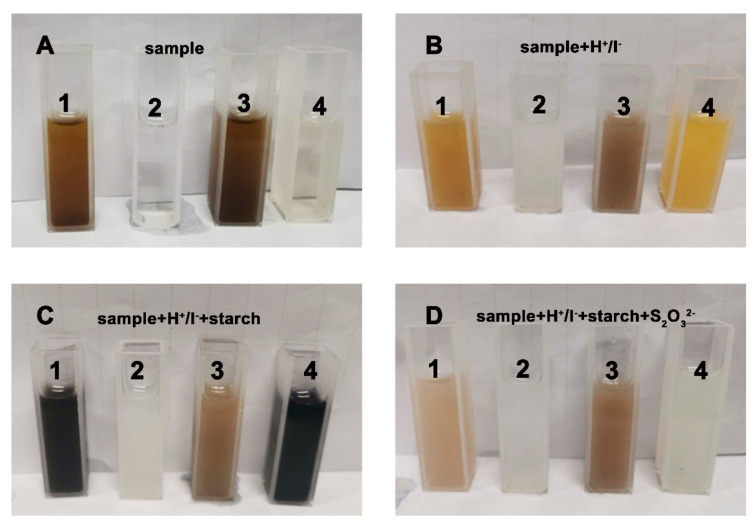
Color changes in (1) PAN/GO/DCDMH, (2) PAN, (3) PAN/GO, and (4) PAN/DCDMH during the iodometric/thiosulfate assay: (**A**) sample suspension; (**B**) sample + H^+^/I^−^ system; (**C**) sample + H^+^/I^−^ + starch system; (**D**) sample + H^+^/I^−^ + starch + S_2_O_3_^2−^ system.

The antibacterial action of *N*-halamine is dependent on the oxidative halogen (i.e., Cl^+^), which serves as an antibacterial site to attack bacteria via physical contact or a Cl^+^ releasing pathway [49]. The presence of the oxidative halogen was indicated through the XPS spectra and EDX analysis. To further confirm the existence of chlorine in the fibrous membrane, the as-electrospun products were examined using an iodometric/thiosulfate assay [50]. The chromaticity evolutions of four products—(1) PAN/GO/DCDMH, (2) PAN, (3) PAN/GO, and (4) PAN/DCDMH—during the assay are shown in Figure 7. In contrast to PAN and PAN/GO, which exhibited no chromaticity evolutions during the iodometric/thiosulfate reaction, PAN/GO/DCDMH and PAN/DCDMH (Figure 7A) oxidized iodide ions into iodine (Figure 7B), allowing a chromogenic reaction by the addition of a starch solution to yield a blue suspension (Figure 7C). The as-formed iodine then reacted with thiosulfate, and the color faded (Figure 7D). The corresponding two oxidation-reduction reactions are indicated below [50]. These color changes confirmed the existence of oxidative halogens in the as-electrospun fibrous membrane, which endow the product with excellent bacteria-killing capability.

N–Cl + 2I^−^ + H^+^ → N-H + I_2_ + Cl^−^

I_2_ + 2S_2_O_3_^2−^ → 2I^−^ + S_4_O_6_^2−^

**Figure 8:**
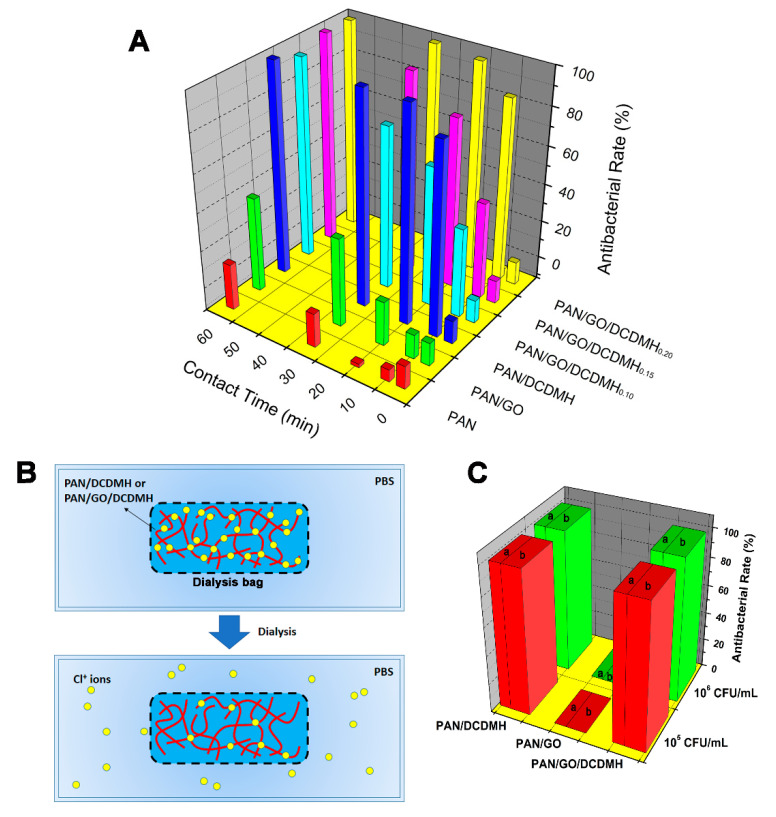
(**A**) Time-kill assay with fibrous membranes against *E. coli*. (**B**) Schematic illustration of dialysis test. (**C**) Antibacterial rate of dialysate solution from PAN/GO, PAN/DCDMH, and PA/GO/DCDMH with different suspension concentrations of (**a**) 2 mg∙mL^−1^ and (**b**) 5 mg∙mL^−1^ against 10^5^ and 10^6^ CFU∙mL^−1^ of *E. coli*.

The antibacterial activity of the as-electrospun fibers was examined using the colony-counting method, with *E. coli* as a typical pathogen [40]. Figure 8 presents the results of the antibacterial kinetic test. The bacteria-killing efficacy of the as-prepared four fibrous membranes—PAN, PAN/GO, PAN/DCDMH, and PAN/GO/DCDMH—against *E. coli* were compared. The pristine PAN displayed no antibacterial activity after 60 h, whereas PAN/GO, PAN/DCDMH, and PAN/GO/DCDMH showed biocidal efficacy, suggesting that the antibacterial function of the electrospun fibers was provided by GO and/or DCDMH. Compared to PAN/GO, PAN/DCDMH and PAN/GO/DCDMH exhibited higher biocidal activity against *E. coli*, indicating DCDMH was more effective at inactivating the bacteria than the GO nanosheets. When three PAN/GO/DCDMH products with different DCDMH loadings (PAN/GO/DCDMH_0.10_, PAN/GO/DCDMH_0.15_, and PAN/GO/DCDMH_0.20_) were compared, the impact of the DCDMH on antibacterial effectiveness was obvious. Clearly, the higher the DCDMH loading, the more active the material will be. Accordingly, it can be concluded that since the antibacterial activity of PAN/GO/DCDMH depends on DCDMH loading, the bacteria-killing ability of the fibrous membrane could be regulated facilely by the DCDMH loading.

To further understand the antibacterial action of PAN/GO/DCDMH, a dialysis test was performed using a dialysis bag with a molecular weight cutoff of 100 Da. The PAN/GO/DCDMH suspension was put into the dialysis bag, which was then immersed in a PBS system. After 2 weeks of shaking (Figure 8B), the chlorine content in the outer water system was examined via iodometric/thiosulfate assay. Active chlorine ions had been released out of the dialysis bag, and the same chromaticity evolution was detected. When the outer water system was examined for its antibacterial effectiveness, the dialysates from the 2 and 5 mg∙mL^−1^ PAN/DCDMH and PA/GO/DCDMH suspensions showed obvious antibacterial activity against both 10^5^ and 10^6^ CFU∙mL^–1^ of *E. coli* (Figure 8C), demonstrating the feasibility of the release-killing mechanism for PAN/GO/DCDMH. These results indicate that PAN/GO/DCDMH inactivates bacteria both on contact and by releasing active chlorine. Accordingly, it is believed that the fibrous membrane has great potential for utilization in water and air purification, biomedical use, textile products, and other antibacterial-associated fields.

## 4. Conclusions

In summary, we fabricated an antibacterial fibrous membrane with controlled nanosizes for combating bacteria without causing drug-resistance, using the electrospinning technique, adjusting the synthesis by tuning the feed ratio of *N*-halamine to GO. After the as-electrospun fibrous membrane was characterized, it was found that both DCDMH and GO played a significant role in killing *E. coli*. More importantly, our antibacterial experiments demonstrated that as a novel antimicrobial agent, the membrane can inactivate *E. coli* either on contact or by releasing active chlorine. We believe this *N*-halamine/GO-functionalized fibrous membrane is capable of combating antibiotic-resistance caused by superbugs and has great potential in antibacterial-associated fields, such as water treatment, air disinfection, medical and healthcare products, textile products, and others.

## Figures and Tables

**Table 1:** The feed ratios of PAN, GO, and DCDMH to DMF in the synthesis of PAN/GO/DCDMH.

Types of Fibrous Membrane	Feed Ratios			
	PAN (g)	GO (g)	DCDMH (g)	DMF (mL)
PAN	1.15	-	-	10
PAN/GO	1.15	0.05	-	10
PAN/DCDMH	1.15	-	0.15	10
PAN/GO_0.05_/DCDMH	1.15	0.05	0.15	10
PAN/GO_0.1_/DCDMH	1.15	0.10	0.15	10
PAN/GO_0.15_/DCDMH	1.15	0.15	0.15	10
PAN/GO/DCDMH_0.1_	1.15	0.05	0.10	10
PAN/GO_0.1_/DCDMH_0.15_	1.15	0.05	0.15	10
PAN/GO_0.1_/DCDMH_0.20_	1.15	0.05	0.20	10

**Table 2:** Elemental information of PAN, PAN/DCDMH, PAN/GO, and PAN/GO/DCDMH detected by EDX spectra.

Elements	PAN	PAN/DCDMH	PAN/GO	PAN/GO/DCDMH
Carbon	+	+	+	+
Oxygen	−	+	+	+
Nitrogen	+	+	+	+
Chlorine	−	+	−	+

“+” presence; “−” absence.

## Data Availability

Not applicable.

## References

[1] Sang Y., Li W., Liu H., Zhang L., Wang H., Liu Z., Ren J., Qu X. (2019). Construction of nanozyme-hydrogel for enhanced capture and elimination of bacteria. Adv. Funct. Mater..

[2] Zhao J., Qu Y., Chen H., Xu R., Yu Q., Yang P. (2018). Self-assembled proteinaceous wound dressings attenuate secondary trauma and improve wound healing in vivo. J. Mater. Chem. B.

[3] Wei T., Yu Q., Chen H. (2019). Responsive and synergistic antibacterial coatings: Fighting against bacteria in a smart and effective way. Adv. Healthc. Mater..

[4] Almeida A., Alves M., Domingues I., Henriques I. (2019). The impact of antibiotic exposure in water and zebrafish gut microbiomes: A 16S rRNA gene-based metagenomic analysis. Ecotoxicol. Environ. Saf..

[5] Liu W., Zhang Y., Zhang Y., Dong A. (2020). Black phosphorus nanosheets counteract bacteria without causing antibiotic resistance. Chem. Eur. J..

[6] Xu M., Song Q., Gao L., Liu H., Feng W., Huo J., Jin H., Huang L., Chai J., Pei Y. (2020). Single-step fabrication of catechol-ε-poly-L-lysine antimicrobial paint that prevents superbug infection and promotes osteoconductivity of titanium implants. Chem. Eng. J..

[7] Wang S., Xu M., Huang K., Zhi J., Sun C., Wang K., Zhou Q., Gao L., Jia Q., Shi H. (2020). Biocompatible metal-free organic phosphorescent nanoparticles for efficiently multidrug-resistant bacteria eradication. Sci. China Mater..

[8] Gao Q., Li P., Zhao H., Chen Y., Jiang L., Ma P. (2017). Methacrylate-ended polypeptides and polypeptoids for antimicrobial and antifouling coatings. Polym. Chem..

[9] Wang X., Li X., Yang X., Lei K., Wang L. (2020). The innovative fabrication of nano-natural antimicrobial agent@polymeric microgels-TiO_2_ hybrid films capable of absorbing UV and antibacterial on touch screen panel. Colloids Surf. B.

[10] Zhou M., Qian Y., Xie J., Zhang W., Jiang W., Xiao X., Chen S., Dai C., Cong Z., Ji Z. (2020). Poly(2-oxazoline)-based functional peptide mimics: Eradicating MRSA infections and persisters while alleviating antimicrobial resistance. Angew. Chem. Int. Ed..

[11] Wang Z., Wang Y., Peng X., He Y., Wei L., Su W., Wu J., Cui L., Liu Z., Guo X. (2019). Photocatalytic antibacterial agent incorporated double-network hydrogel for wound healing. Colloids Surf. B.

[12] Dong A., Sun Y., Lan S., Wang Q., Cai Q., Qi X., Zhang Y., Gao G., Liu F., Harnoode C. (2013). Barbituric acid-based magnetic *N*-halamine nanoparticles asrecyclable antibacterial agents. ACS Appl. Mater. Interfaces.

[13] Dong A., Lan S., Huang J., Wang T., Zhao T., Xiao L., Wang W., Zheng X., Liu F., Gao G. (2011). Modifying Fe_3_O_4_-functionalized nanoparticles with *N*-halamine and their magnetic/antibacterial properties. ACS Appl. Mater. Interfaces.

[14] Dong A., Wang Y., Gao Y., Gao T., Gao G. (2017). Chemical insights into antibacterial *N*-halamines. Chem. Rev..

[15] Dong A., Huang Z., Lan S., Wang Q., Bao S., Siriguleng S., Zhang Y., Gao G., Liu F., Harnoode C. (2014). *N*-Halamine-decorated polystyrene nanoparticles based on 5-allylbarbituricacid: From controllable fabrication to bactericidal evaluation. J. Colloid Interface Sci..

[16] Natan M., Gutman O., Lavi R., Margel S., Banin E. (2015). Killing mechanism of stable *N*-halamine cross-linked polymethacrylamide nanoparticles that selectively target bacteria. ACS Nano.

[17] Bai R., Zhang Q., Li L., Li P., Wang Y., Simalou O., Zhang Y., Gao G., Dong A. (2016). *N*-Halamine-containing electrospun fibers kill bacteria via a contact/release co-determined antibacterial pathway. ACS Appl. Mater. Interfaces.

[18] Xin Q., Shah H., Nawaz A., Xie W., Akram M.Z., Batool A., Tian L., Jan S.U., Bodula R., Guo B. (2018). Antibacterial carbon-based nanomaterials. Adv. Mater..

[19] Rasool K., Helal M., Ali A., Ren C., Gogotsi Y., Mahmoud K.A. (2016). Antibacterial activity of Ti_3_C_2_Tx MXene. ACS Nano.

[20] Selim M.S., Mo P.J., Hao Z., Fatthallah N.A., Chen X. (2020). Blade-like structure of graphene oxide sheets decorated with cuprous oxide and silicon carbide nanocomposites as bactericidal materials. J. Colloid Interface Sci..

[21] Lu B., Zhu G., Yu C., Chen G., Zhang C., Zeng X., Chen Q., Peng Q. (2002). Functionalized graphene oxide nanosheets with unique three-in-one properties for efficient and tunable antibacterial applications. Nano Res..

[22] Zou X., Zhang L., Wang Z., Luo Y. (2016). Mechanisms of antimicrobial activities of graphene materials. J. Am. Chem. Soc..

[23] Abadikhah H., Kalali E.N., Khodi S., Xu X., Agathpoulos S. (2019). Multifunctional thin-film nanofiltration membrane incorporated with reduced graphene oxide@TiO_2_@Ag nanocomposites for high desalination performance, dye retention, and antibacterial properties. ACS Appl. Mater. Interfaces.

[24] Ghosh S., Goudar V.S., Padmalekha K.G., Bhat S.V., Indi S.S., Vasan H.N. (2012). ZnO/Ag nanohybrid: Synthesis, characterization, synergistic antibacterial activity and its mechanism. RSC Adv..

[25] Aksoy I., Küçükkeçeci H., Sevgi F., Metin Ö., Patir I.H. (2020). Photothermal antibacterial and antibiofilm activity of black phosphorus/gold nanocomposites against pathogenic bacteria. ACS Appl. Mater. Interfaces.

[26] Borjihan Q., Dong A. (2020). Design of nanoengineered antibacterial polymers for biomedical applications. Biomater. Sci..

[27] Robert B., Nallathambi G. (2020). A concise review on electrospun nanofibers/nanonets for filtration of gaseous and solid constituents (PM_2.5_) from polluted air. Colloid Interface Sci. Commun..

[28] Liu C., Shen J., Yeung K.W.K., Tjong S.C. (2017). Development and antibacterial performance of novel polylactic acid-graphene oxide-silver nanoparticle hybrid nanocomposite mats prepared by electrospinning. ACS Biomater. Sci. Eng..

[29] Adhikari S.P., Awasthi G.P., Lee J., Park C.H., Kim C.S. (2016). Synthesis, characterization, organic compound degradation activity and antimicrobial performance of g-C_3_N_4_ sheets customized with metal nanoparticles-decorated TiO_2_ nanofibers. RSC Adv..

[30] Choi J., Yang B.Y., Bae G., Jung J.H. (2015). Heral extract incorporated nanofiber fabricated by an electrospinning technique and its application to antimicrobial air filtration. ACS Appl. Mater. Interfaces.

[31] Daristotle J.L., Behrens A.M., Sandler A.D., Kofinas P. (2016). A review of the functional principles and applications of solution blow spinning. ACS Appl. Mater. Interfaces.

[32] Ren H., Du Y., Su Y., Guo Y., Zhu Z., Dong A. (2018). A review on recent achievements and current challenges in antibacterial electrospun *N*-halamines. Colloid Interface Sci. Commun..

[33] Rieger K.A., Birch N.P., Schiffman J.D. (2013). Designing electrospun nanofiber mats to promote wound healing-a review. J. Mater. Chem. B.

[34] Mukhejee M., De S. (2018). Antibacterial polymeric membranes: A short review. Environ. Sci. Water Res. Technol..

[35] Lu T., Cui J., Qu Q., Wang Y., Zhang J., Xiong R., Ma W., Huang C. (2021). Multistructured electrospun nanofibers for air filtration: A review. ACS Appl. Mater. Interfaces.

[36] Li P., Gao Y., Sun Z., Chang D., Gao G., Dong A. (2017). Synthesis, Characterization, and bactericidal evaluation of chitosan/guanidine functionalized graphene oxide composites. Molecules.

[37] Li H., Liu K., Williams G.R., Wu J., Wu J., Wang H., Niu S., Zhu L. (2018). Dual temperature and pH responsive fibrous formulations prepared by electrospinning. Colloid Surf. B.

[38] Lan S., Sheng X., Lu Y., Li C., Zhao S., Liu N. (2018). Modification of antibacterial ZnO nanorods with CeO_2_ nanoparticles: Role of CeO_2_ in impacting morphology and antibacterial activity. Colloid Interface Sci. Commun..

[39] Ahmed A.E.I., Hay J.N., Bushell M.E., Wardell J.N., Cavalli G. (2008). Biocidal polymers (II): Determination of biological activity of novel *N*-halamine biocidal polymers and evaluation for use in water filters. React. Funct. Polym..

[40] Lan S., Lu Y., Li C., Zhao S., Liu N., Sheng X. (2019). Sesbania gum-supported hydrophilic electrospun fibers containing nanosilver with superior antibacterial activity. Nanomaterials.

[41] Gao Y., Song N., Liu W., Dong A., Wang Y., Yang Y. (2019). Construction of antibacterial *N*-halamine polymer nanomaterials capable of bacterial membrane disruption for efficient anti-infective wound therapy. Macromol. Biosci..

[42] Upadhyay R.K., Kumar A. (2019). Effect of particle weight concentration on the lubrication properties of graphene based epoxy composites. Colloid Interface Sci. Commun..

[43] Borjihan Q., Zhang Z., Zi X., Huang M., Chen Y., Zhang Y., Dong A. (2020). Pyrrolidone-based polymers capable of reversible iodine capture for reuse in antibacterial applications. J. Hazard. Mater..

[44] Chen Z., Sun Y. (2006). *N*-Halamine-based antimicrobial additives for polymers: Preparation, characterization, and antimicrobial activity. Ind. Eng. Chem. Res..

[45] Bai R., Kang J., Simalou O., Liu W., Ren H., Gao T., Gao Y., Chen W., Dong A., Jia R. (2018). Nover N-Br bond-containing *N*-halamine nanofibers with antibacterial activities. ACS Biomater. Sci. Eng..

[46] Borjihan Q., Yang J., Song Q., Gao L., Xu M., Gao T., Liu W., Li P., Li Q., Dong A. (2019). Povidone-iodine-functionalized fluorinated copolymers with dual-functional antibacterial and antifouling activities. Biomater. Sci..

[47] Upadhyay R.K., Kumar A. (2020). Enzyme-mimetic activity of sugar cane juice stabilized CuO nanospheres and CuO/GO nanocomposite: Green synthesis and applications. Colloid Interface Sci. Commun..

[48] Jie Z., Yan X., Zhao L., Worley S.D., Liang J. (2014). Eco-friendly synthesis of regenerable antimicrobial polymeric resin with *N*-halamine and quaternary ammonium salt groups. RSC Adv..

[49] Chen Z., Luo J., Sun Y. (2007). Biocidal efficacy, biofilm-controlling function, and controlled release effect of chloromelamine-based bioresponsive fibrous Materials. Biomaterials.

[50] Badrossamay M.R., Sun G. (2009). Graft polymerization of N-tert-butylacrylamide onto polypropylene during melt extrusion andbiocidal properties of its products. Polym. Eng. Sci..

